# Reunion of a Divided Nerve

**Published:** 1845-01

**Authors:** 


*Reunion of a divided Nerve.*—Dr. Oke relates, in the Provincial Medical and Surgical Journal, July 24th, 1844, the following interesting fact in a physiological point of view:

In cutting through the muscles in an operation for necrosis of the humerus, the musculo-cutaneous nerve was unavoidably divided, which occasioned the instantaneous dropping of the hand. This appeared at first to be an unfortunate result, as it was feared it might occasion the permanent loss of the hand and deprive the patient of the power of writing, for he could use his pen tolerably well.

To make up for so serious a deprivation in some degree, as soon as the state of the right arm would admit of it, he was sent to a schoolmaster to be taught to write with his left hand, which, in about four months, he accomplished; but at the end of this time, I was gratified to observe a returning power in the right hand. The power gradually increased; and in the course of a few weeks he completely regained its use, and thus was enabled to write with both hands.

This was an important fact in physiology, inasmuch as it showed that the trunk of a nerve, though it be divided and its ends kept apart for a considerable time, might eventually unite and regain its normal functions.

*American Journal Medical Sciences.*

